# Antimicrobial Peptide Resistance Mechanisms of Gram-Positive Bacteria

**DOI:** 10.3390/antibiotics3040461

**Published:** 2014-10-13

**Authors:** Kathryn L. Nawrocki, Emily K. Crispell, Shonna M. McBride

**Affiliations:** Department of Microbiology and Immunology, Emory University School of Medicine, 1510 Clifton Rd, Atlanta, GA 30322, USA; E-Mails: knawroc@emory.edu (K.L.N.); ecrispell@emory.edu (E.K.C.)

**Keywords:** *Clostridium difficile*, antimicrobial, antimicrobial peptide, AMP, resistance

## Abstract

Antimicrobial peptides, or AMPs, play a significant role in many environments as a tool to remove competing organisms. In response, many bacteria have evolved mechanisms to resist these peptides and prevent AMP-mediated killing. The development of AMP resistance mechanisms is driven by direct competition between bacterial species, as well as host and pathogen interactions. Akin to the number of different AMPs found in nature, resistance mechanisms that have evolved are just as varied and may confer broad-range resistance or specific resistance to AMPs. Specific mechanisms of AMP resistance prevent AMP-mediated killing against a single type of AMP, while broad resistance mechanisms often lead to a global change in the bacterial cell surface and protect the bacterium from a large group of AMPs that have similar characteristics. AMP resistance mechanisms can be found in many species of bacteria and can provide a competitive edge against other bacterial species or a host immune response. Gram-positive bacteria are one of the largest AMP producing groups, but characterization of Gram-positive AMP resistance mechanisms lags behind that of Gram-negative species. In this review we present a summary of the AMP resistance mechanisms that have been identified and characterized in Gram-positive bacteria. Understanding the mechanisms of AMP resistance in Gram-positive species can provide guidelines in developing and applying AMPs as therapeutics, and offer insight into the role of resistance in bacterial pathogenesis.

## 1. Introduction

Antimicrobial peptides (AMPs) and the bacterial resistance mechanisms against them have been co-evolving for eons. A diverse array of life forms can produce AMPs, which can be used to promote immune defenses, nutrient acquisition or elimination of rival organisms from the environment. As a result, AMPs are found in a multitude of environments, ranging from mammalian tissues to soil and aquatic environments. This ubiquitous presence of AMPs in the environment provides strong selective pressure to drive the development of bacterial resistance against these peptides. 

AMPs are typically small, charged, amphipathic molecules that can be produced in a variety of structures. Though structurally diverse, most AMPs work by interacting with the bacterial cell surface, followed by disruption of cellular integrity. Accordingly, the majority of bacterial resistance mechanisms function by limiting the interaction of AMPs with the bacterial cell surface. Mechanisms of AMP resistance include trapping or sequestering of peptides, outright destruction of AMPs by proteolysis, removal of AMPs from the cell via active transport, and structural modification of the cell surface to avoid interaction with AMPs. Many of these resistance mechanisms are upregulated in response to AMPs, allowing the bacteria to adaptively counter the effects of AMPs. Loss of these resistance mechanisms can impair the ability of bacteria to colonize plant or animal hosts and can attenuate virulence for many pathogens. Mechanisms of resistance may evolve specifically within a bacterial lineage or be genetically transferred from other AMP-resistant organisms.

In this review, we evaluate the available literature on Gram-positive bacterial resistance mechanisms to antimicrobial peptides. This review highlights methods of AMP resistance based on mode of action and location within the Gram-positive bacterial cell. We begin with an overview of resistance mechanisms that act on AMPs extracellularly, and then discuss bacterial cell surface alterations. Finally, we consider removal of AMPs from the bacterial cell via transport.

## 2. Extracellular Mechanisms of Resistance: Enzymatic Degradation and AMP Blocking

**Figure 1:**
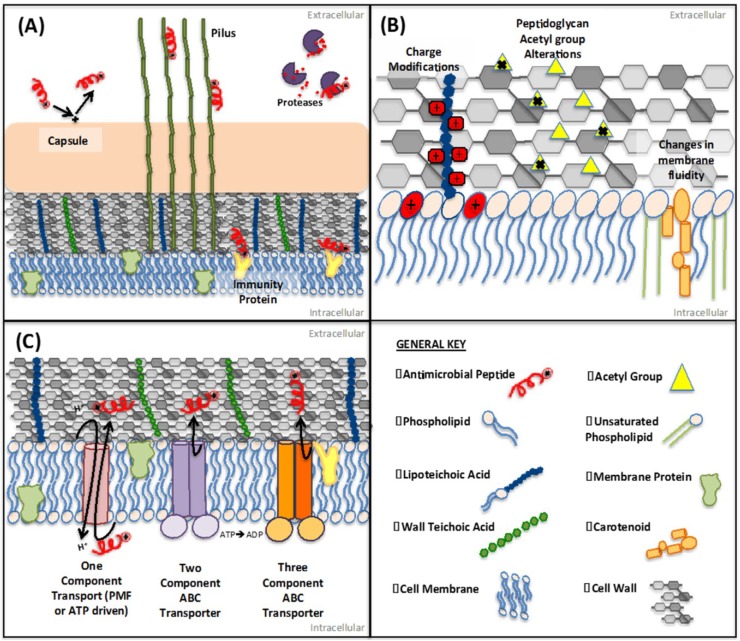
Overview of Antimicrobial Peptide Resistance Mechanisms in Gram-Positive Bacteria. (**A**) Extracellular mechanisms of AMP resistance include peptide degradation by secreted proteases, AMP sequestration by secreted or membrane associated protein (e.g., pili, immunity proteins, M proteins), or blocking by capsule polysaccharides; (**B**) Cell wall and membrane modifications include: Alteration of charge by lysination of the phospholipid head groups or d-alanylation of the lipoteichoic backbone, modification of the cell wall by deacetylation of N-acetylglucosamine or O-acetylation of N-acetylmuramyl residues, and alterations in membrane fluidity by phospholipid tail saturation or carotenoid additions; (**C**) Transport mechanisms of antimicrobial efflux from the cell include: ATP-driven ABC transporters composed of a single, double, or triple protein pump and involve a supplementary immunity protein, or single protein transporters driven by proton motive force.

The initial site of AMP interaction is at the bacterial cell surface. As a result, extracellular mechanisms of AMP inactivation have evolved as a first line of defense to minimize damage to the bacterial cell. Extracellular AMP resistance mechanisms have arisen in two main forms: enzymatic inactivation and sequestration (see Table 1 and Figure 1). The majority of these direct targeting mechanisms have evolved to recognize cationic AMPs. Cationic AMPs are positively charged peptides that may differentially target negatively charged moieties on the outer cell envelope, including teichoic acids, lipid II, and phosphatidylglycerol [1,2,3].

### 2.1. Extracellular Proteases

The degradation of AMPs by proteases is a mechanism of resistance found in many Gram-positive species, including *Enterococcus faecalis*, *Staphylococcus aureus*, and *Staphylococcus epidermidis* [4,5,6]. AMP-degrading proteases generally have broad substrate specificity, are typically found in mammalian pathogens, and include both metallopeptidases and cysteine proteases [7,8]. This section will present several examples of AMP-degrading proteases produced by Gram-positive bacteria and detail their effects on resistance.

AMP-degrading proteases are often secreted by bacteria into their surrounding extracellular environments. Gelatinase, an extracellular metallopeptidase produced by some strains of the opportunistic pathogen *E. faecalis*, cleaves the human cathelicidin, LL-37, resulting in the loss of antimicrobial activity *in vitro* [4]. The production of gelatinase by *E. faecalis* is associated with bacterial dissemination in animal models of disease and with increased incidence of dental caries in humans [9,10]. One example of a secreted protease made by *S. aureus* that confers AMP resistance is the aureolysin enzyme [5]. Aureolysin can hydrolyze the C-terminal bactericidal domain of LL-37, rendering the AMP inactive [11]. An infection model using human macrophages revealed that aureolysin contributes to Staphylococcal persistence within the phagosomal compartment [12], an environment that contains high levels of the antimicrobial peptide, LL-37 [13]. Additionally, some species of Staphylococci possess proteases that combat anionic AMPs such as dermcidin, a negatively charged peptide secreted by human sweat glands [14]. SepA (or SepP1) made by *S. epidermidis*, is a secreted metalloprotease that can cleave and inactivate dermcidin [6,15]. The SepA protease appears to specifically target dermcidin *in vitro* [6,16]. 

Gram-positive proteases are also capable of targeting AMPs at the bacterial surface. SpeB is a cysteine proteinase secreted by the pathogenic bacterium *Streptococcus pyogenes* [17]. SpeB has broad substrate specificity and cleaves AMPs, such as LL-37, and other host proteins such as fibrin, immunoglobulins, and other immune modulators [4,18,19,20,21]. In an example of adaptive resistance, SpeB was found to complex with the host α_2_-macroglobulin (α_2_M) proteinase inhibitor during infection [22]. The catalytically active SpeB-α_2_M complexes are retained on the bacterial cell surface by association with the *S. pyogenes* G-related α_2_M-binding protein (GRAB) [22,23]. The SpeB-α_2_M complex has higher proteinase activity against LL-37, relative to free SpeB, and reduces killing of *S. pyogenes* *in vitro* [22]. 

**Table 1:** Summary of Gram-positive Antimicrobial Peptides (AMP) Resistance Mechanisms.

Name	Mechanism of Action	Antimicrobial Resistance	Organisms	Reference
*AMP Degradation*				
Aureolysin	Protease	LL-37	*S. aureus*	[5,11]
Gelatinase	Protease	LL-37	*E. faecalis*	[4,10]
SepA	Protease	dermcidin	*S. epidermidis*	[6,16]
SpeB	Protease	LL-37	*S. pyogenes*	[4,21,22]
*Sequestration/Competition for AMP target*				
M Protein	Binding at surface	LL-37	*S. pyogenes*	[24]
PilB	Binding at surface	cathelicidins	*S. agalactiae*	[25]
SIC	Extracellular binding	α-defensins, LL-37, lysozyme	*S. pyogenes*	[26,27]
Staphylokinase	Extracellular binding	Cathelicidin, defensins	*S. aureus*	[28,29]
LciA	Binding at surface	Lactococcin A	*L. lactis*	[30,31]
Capsule	Binding/shielding	Polymyxin B, HNP-1	*S. pneumoniae*	[32]
Exopolysaccharide	Shielding/ Sequestration	LL-37, hBD-3, dermcidin	*S. epidermidis*	[33,34,35]
LanI lipoproteins	Binding or competition	lantibiotics	*L. lactis*, *B. subtilis*, other lantibiotic producers	[36,37,38]
*Cell Surface Modifications*				
DltABCD	d-alanylation of teichoic acids	daptomycin, vancomycin, nisin, defensins, protegrins	*S. aureus*, *L. monocytogenes*, *B. cereus*, *C. difficile*, *S. pyogenes*, *S. agalactiae*, *B. anthracis*, *S. suis*	[2,39,40,41,42,43,44,45]
MprF	Lysylation of phoshatidylglycerol	defensins, thrombin-induced platelet microbicidal protein	*S. aureus*, *L. monocytogenes*, *B. anthracis*, *M. tuberculosis*	[46,47,48,49,50]
OatA	Peptidoglycan O-acetylase	lysozyme	*S. aureus*, *S. epidermidis, S. lugdunensis*, *E. faecalis*, *L. monocytogenes*	[51,52,53,54]
PdgA	Peptidoglycan *N*-acetylglucosamine deacetylase A	lysozyme	*S. pneumoniae*, *E. faecalis*, *S. suis*, *L. monocytogenes*, *B. anthracis*	[55,56,57,58]
NamH	*N*-acetylmuramic acid hydroxylase	lysozyme	*M. smegmatis*	[59]
*AMP Efflux*				
*One-component transporter*				
LmrB	ABC transporter	LsbA/LsbB	*L. lactis*	[60]
QacA	ABC transporter/alteration of membrane structure	thrombin-induced platelet microbicidal protein (tPMP)	*S. aureus*	[61]
*BceAB type*				
AnrAB	ABC transporter	nisin, gallidermin, bacitracin, β-lactams	*L. monocytogenes*	[62,63]
BceAB	ABC transporter	Bacitracin ^a^, actagardine, mersacidin, plectasin	*B. subtilis ^a^*, *S. mutans*	[64,65,66,67,68]
BraAB	ABC transporter	nisin, nukacin ISK-1, bacitracin	*S. aureus*	[69]
PsdAB	ABC transporter	nisin, enduracidin, gallidermin, subtilin	*B. subtilis*	[66]
MbrAB	ABC transporter	bacitracin	*S. mutans*	[35]
SP0812-SP0813	ABC transporter	bacitracin, vancoresmycin	*S. pneumoniae*	[70]
SP0912-SP0913	ABC transporter	bacitracin, lincomycin, nisin	*S. pneumoniae*	[71]
VraDE	ABC transporter	bacitracin, nisin, nukacin ISK-1	*S. aureus*	[69,72,73,74,75,76]
VraFG	ABC transporter	nisin, colistin, bacitracin, vancomycin, indolicidin, LL-37, hBD3	*S. aureus*, *S. epidermidis*	[69,72,75,77,78,79]
YsaCB	ABC transporter	nisin	*L. lactis*	[80]
*BcrAB type*				
BcrAB(C)	ABC transporter	bacitracin	*B. licheniformis*	[81]
BcrAB(D)	ABC transporter	bacitracin	*E. faecalis*	[82,83]
*LanFEG type*				
As-48EFG(H)	ABC transporter	AS-48	*E. faecalis*	[84]
CprABC	ABC transporter	nisin, galidermin, other lantibiotics	*C. difficile*	[85,86]
EpiFEG(H)	ABC transporter	epidermin, gallidermin	*S. epidermidis*	[87]
LtnFE(I)	ABC transporter	lacticin 3147	*L. lactis*	[88,89]
McdFEG	ABC transporter	macedocin	*S. macedonicus*	[90]
MrsFGE	ABC transporter	mersacidin	*Bacillus sp. HIL Y-84*, *54728*	[91,92]
MutFEG	ABC transporter	mutacin II	*S. mutans*	[93]
NisFEG(I)	ABC transporter	nisin	*L. lactis*	[37,94]
NukFEG(H)	ABC transporter	nukacin	*S. warneri*	[95,96]
SboFEG	ABC transporter	salivaricin B	*S. salivarius*	[97]
ScnFEG	ABC transporter	streptococcin A-FF22	*S. pyogenes*	[98]
SmbFT	ABC transporter	Smb, haloduracin	*S. mutans*	[99]
SpaFEG	ABC transporter	subtilin	*B. subtilis*	[36,100]

^a^ Confers only bacitracin resistance in *B. subtilis*.

### 2.2. Protein-Mediated Sequestration

Sequestration is another extracellular mechanism of AMP resistance [24,25,26,27,28,29,101]. Some Gram-positive bacteria produce extracellular or surface-linked proteins that directly bind to AMPs and block access to the cell membrane. Mechanisms of protein-mediated AMP sequestration vary between species and strains. We have highlighted specific examples of AMP sequestration mechanisms identified amongst strains of *S. pyogenes*, *S. aureus*, *Streptococcus agalactiae*, and *Lactococcus lactis*.

Proteins that inhibit AMP activity through binding can be secreted into the extracellular environment to inhibit contact of bactericidal peptides with the cellular surface. For example, the Streptococcal inhibitor of complement (SIC) produced by *S. pyogenes* is a hydrophilic, secreted protein that sequesters many AMPs, thereby preventing them from reaching cell-surface targets [102]. SIC binds to α-defensins, LL-37, and lysozyme, neutralizing the AMPs and inhibiting their bactericidal activity against *S. pyogenes* [27,102,103]. SIC production promotes bacterial survival *in vitro* and increases the virulence of *S. pyogenes* in animal models of disease [26,104]. Staphylokinase secretion by *S. aureus* is another example of an extracellular AMP resistance mechanism. Production of the staphylokinase protein by *S. aureus* occurs through the lysogenic conversion of the hlb β-hemolysin toxin gene by a bacteriophage harboring the *sak* gene [105,106,107]. Staphylokinase binds the murine cathelicidin mCRAMP *in vivo* and also complexes with human defensins HNP-1 and HNP-2 to reduce their bactericidal effects [28,29]. Studies of staphylokinase binding suggest that the staphylokinase-cathelicidin complex promotes host tissue invasion by activating the conversion of plasminogen to the host extracellular matrix-degrading enzyme, plasmin, although the role this conversion plays in Staphylococcal virulence remains unclear [29,101,108].

Proteins attached to the cellular surface can also bind AMPs to prevent contact with cell-associated targets. Examples of such proteins include the M1 protein of *S. pyogenes* and the pilus subunit, PilB of *S. agalactiae*. M1 of *S. pyogenes* can be found on the surface of most clinical isolates and has been linked to both host tissue adherence and invasive disease [109]. A hyper-variable extracellular portion of the M1 protein was shown to bind LL-37 and prevent the AMP from reaching the cell membrane [24]. The sequestration of LL-37 by M1 also promotes Streptococcal survival in neutrophil extracellular traps (NETs) by reducing LL-37 activity [24]. Like the M proteins of *S. pyogenes*, pili are also associated with invasive disease and promotion of host cell adherence by *S. agalactiae* [110,111]. Pili are large, filamentous, multimeric protein complexes expressed on the cell surface of *S. agalactiae* and other bacteria. Expression of the Streptococcal pilin subunit, PilB, promotes association of LL-37 with the bacterial cell surface and correlates with increased resistance to the murine cathelicidin mCRAMP *in vitro* [25]. In addition, pilB mutants of *S. agalactiae* (GBS) exhibit reduced fitness relative to wild-type strains in murine infection models [25]. These data suggest that in addition to the adhesin properties of pili, pilus-mediated binding of AMPs also contributes to *S. agalactiae* virulence within the host. 

Another family of membrane-associated AMP resistance proteins encompasses the LanI immunity proteins of some bacteriocin producer strains. LanI proteins are typically encoded near a bacteriocin biosynthetic operon and provide protection against the bacteriocin made by the producer bacterium [112,113]. LanI-type immunity proteins are lipoproteins that anchor to the bacterial cell surface and confer resistance by either binding directly to AMPs or outcompeting AMPs by binding directly to the cellular target [114,115,116,117]. The LanI lipoproteins often work in concert with LanFEG transporters, possibly acting as substrate-binding partners for specific lantibiotics. The best characterized of the transporter-associated LanI proteins are the NisI and SpaI lipoproteins found in strains of *L. lactis* and *Bacillus subtilis*, respectively [36,37,118] (described in transporter section). But, several lantibiotic producers encode only a LanI immunity protein and do not encode an apparent LanFEG transporter (e.g., PepI of *S. epidermidis* [119], lactocin S [120] of *L. sakei* and epicidin 280 of *S. epidermidis* [121]). In these systems, the LanI lipoprotein confers full immunity to the associated lantibiotic. Though some LanI structures have been characterized, LanI lipoproteins generally have low, if any, homology to one another [116,122]. Thus, it is unclear if mechanism of action for LanI-mediated immunity is conserved between different LanI lipoproteins.

### 2.3. Inhibition of AMP Activity by Surface-Associated Polysaccharides

Extracellular polysaccharide production has long been recognized as a factor that promotes both virulence and host colonization by many bacteria [123,124,125]. Extracellular polysaccharides are composed of structurally diverse polymers that are enzymatically produced by some Gram-positive species [126,127]. Extracellular polysaccharides that are attached to the cellular surface through covalent linkages with the cell wall are known as capsules (capsular polysaccharide, or CPS), while loosely attached polymers are referred to as exopolysaccharides, or EPS [128,129,130]. Polysaccharide-mediated AMP resistance is thought to occur by shielding the bacterial membrane via binding or electrostatic repulsion of AMPs [34,131].

The production of capsular polysaccharides provides resistance to a variety of AMPs and other antimicrobials and can allow some bacteria to evade host detection. Capsule-AMP binding can be mediated by the electrostatic interaction of positively charged AMPs with the negatively charged polysaccharide capsule [32]. For example, free capsular extracts from *Streptococcus pneumoniae* bind both polymyxin B and the α-defensin HNP-1, preventing these AMPs from reaching the cell membrane and promoting bacterial survival *in vitro*. Additionally, both polymyxin B and HNP-1 promote release of the capsule from *S. pneumoniae* without loss of cell viability, suggesting that capsule release may be a mechanism of AMP resistance by sequestering AMPs away from the bacterial cell surface [32]. In another example, production of the exopolysaccharide intercellular adhesion, PIA, by *S. epidermidis* reduces killing by human defensin hBD-3, cathelicidin (LL-37), and the anionic AMP dermcidin. PIA is hypothesized to shield the bacterial membrane from the effects of AMPs [33,34]. Predictably, PIA production is associated with *S. epidermidis* virulence in multiple animal infection models [132,133]. However, while many exopolysaccharide capsules can provide resistance to AMPs, this protection is not universal to all capsule-producing Gram-positive bacteria [134,135,136].

## 3. Membrane and Cell Wall Modifications

The bacterial cell wall and membrane comprise a major target for the bactericidal activity of AMPs [137,138,139]. Bacteria frequently modify cell surface components to counter the effects of AMPs by reducing the net negative charge of the cell, altering membrane fluidity, or directly modifying AMP targets [140,141,142]. 

### 3.1. Repulsion of AMPs

Many AMPs target bacterial cells through electrostatic interactions with the cell surface [137,138,139]. The net charge of the bacterial cell surface is generated by anionic components of the cell membrane and cell wall, such as phospholipids and teichoic acids [143,144,145]. In turn, positively charged AMPs are attracted to the negatively charged bacterial cell surface [144,145]. Hence, a broad strategy of resistance to positively charged AMPs is to alter the components on the cell surface to decrease the net negative charge of the cell, thereby limiting the electrostatic interaction of AMPs with the bacterial cell surface. 

One component of the bacterial cell membrane that carries a negative charge is phosphatidylglycerol [144,145]. But in many Gram-positive bacteria, the negative charge on phosphatidylglycerol can be masked via the addition of a positively charged amino acid by the multipeptide resistance factor protein, MprF [146,147]. MprF is an intergral lysyl-phosphatidylglycerol synthetase that synthesizes and translocates aminoacylated-phosphatidylglycerol to the external membrane layer of the bacterial cell. MprF synthases were initially found to incorporate a positively charged lysine into phosphatidylglycerol (Lys-PG), decreasing the net negative charge on the bacterial membrane. In *S. aureus*, *Listeria monocytogenes*, *E. faecalis*, *Enterococcus faecium*, *B. subtilis*, and *Bacillus anthracis*, the aminoacylation of phosphatidylglycerol by MprF confers resistance to positively charged AMPs [47,48,49,148,149,150]. Additionally, an MprF homolog is present in *Mycobacterium tuberculosis*, which also confers resistance to positively charged AMPs*.* This MprF homolog, LysX, carries out the same functions as MprF, with the addition of a lysyl-tRNA synthetase activity [46,151]. Lysinylation of phosphatidylglycerol confers resistance to a broad spectrum of AMPs, including human defensins, gallidermin, nisin, lysozyme, daptomycin, polymyxin B, and vancomycin (Table 1) [46,150,151,152,153,154,155,156,157,158,159]. In addition to lysine modifications, some MprF orthologs can modify membrane phosphatidylglycerol with multiple amino acids, including alanine and arginine [149,160]. The enhanced antimicrobial resistance provided by aminoacylation of phosphatidylglycerol is also associated with increased virulence for several Gram-positive pathogens [46,48,49,161,162].

The Dlt pathway is another enzymatic mediator of AMP resistance that has been identified and studied in many Gram-positive genera including *Staphylococcus*, *Listeria*, *Enterococcus*, *Bacillus*, *Clostridium*, *Streptococcus*, and *Lactobacillus* [2,40,41,42,43,44,45,163,164,165,166,167,168]. The enzymatic functions of the DltABCD proteins lead to the d-alanylation of teichoic acids and lipoteichoic acids of the cell wall [169]. The addition of d-alanine to the backbone of teichoic acids can mask the negative charge present along these glycopolymers, thereby leading to increased surface charge and lower attraction of positively charged antimicrobials [169]. Similar to MprF, d-alanylation of teichoic acids by the Dlt pathway leads to a global change in charge distribution across the cell surface, allowing resistance to a broad range of cationic AMPs including vancomycin, nisin, gallidermin daptomycin, polymyxin B, lysozyme, and cathelicidins [2,39,141,163,166,170,171,172].

In addition to charge modification of teichoic acids, high-resolution microscopy of Group B *Streptococcus* revealed that d-alanylation could increase cell wall density, leading to increased surface rigidity [173]. Accordingly, d-alanylation may confer resistance to AMPs both by reducing the electrostatic interactions between AMPs and the cell surface and by decreasing the permeability of the cell wall [173]. As AMPs are ubiquitous within animals, d-alanylation of the cell wall can affect host colonization for pathogens and non-pathogenic species [41,152,164,174,175].

### 3.2. Target Modification

The cell wall is a common antimicrobial target for Gram-positive organisms. As a result, bacteria have evolved multiple modifications that limit antimicrobial targeting of the cell wall. Lysozyme, or *N*-acetylmuramide glycanhydrolase, an antimicrobial enzyme, is an important component of the host innate immune defense. Lysozyme is cationic at physiological pH, which facilitates its interaction with negatively charged bacterial surfaces. The cationic and muramidase activities of lysozyme directly target the bacterial peptidoglycan, the primary constituent of the cell wall [176]. The muramidase domain of lysozyme hydrolyzes the β-1,4 linkages between *N*-acetylglucosamine and *N*-acetylmuramic acid of peptidoglycan, leading to the breakdown of the peptidoglycan macromolecular structure and eventual lysis of the cell [177,178,179]. As a result, bacterial resistance mechanisms have evolved to counter both the muramidase and cationic activities of lysozyme. In this section, we detail the mechanisms by which peptidoglycan is modified to limit lysozyme activity. 

Two peptidoglycan modifiers that contribute to AMP resistance in some Gram-positive bacteria are the enzymes PgdA and OatA. It is proposed that the modifications made by both of these enzymes lead to steric hindrance between AMPs and the cell surface, thereby limiting the contact between lysozyme and its target [180]. PgdA deacetylates *N*-acetylglucosamine residues of peptidoglycan, generating a less favorable substrate for lysozyme [181,182,183,184]. PgdA was first implicated as a peptidoglycan deacetylase in the respiratory pathogen *S. pneumoniae*. PdgA and other peptidoglycan deacetylase orthologs have been shown to contribute to AMP resistance in many bacteria, including *Enterococcus*, *Streptococcus*, *Listeria* and *Bacillus* species [55,56,57,58,180,183,185]. Moreover, deacetylation of peptidoglycan enhances colonization and virulence in several pathogens, including *E. faecalis*, *L. monocytogenes* and *S. pneumoniae* [185,186,187]*.* As *N*-acetylglucosamine deacetylases are encoded within the genomes of most Gram-positive bacteria, these enzymes likely contribute to lysozyme and host colonization in many more species. 

OatA (also known as Adr in *S. pneumoniae*) is another type of peptidoglycan modifying enzyme found in Gram-positive bacteria that confers resistance to lysozyme [188,189,190]. OatA performs *O*-acetylation at the C6-OH group of *N*-acetylmuramyl residues in peptidoglycan [188,189,190]. *O*-acetylation of *N*-acetylmuramyl residues is thought to prevent lysozyme from interacting with the β-1,4 linkages of peptidoglycan by steric hindrance [180]. OatA and orthologous proteins have been characterized in *Staphylococcus*, *Enterococcus*, *Lactococcus*, *Bacillus*, *Streptococcus* and *Listeria* species [51,52,54,58,180,187,191]. Like deacetylation mechanisms, *O*-acetylation of peptidoglycan is likely to be widespread among Firmicutes and has been noted to contribute to virulence in animal models of infection [52,54,187,190,192].

A peptidoglycan modifier unique to *Mycobacterium* is the enzyme NamH (*N*-acetylmuramic acid hydroxylase). NamH hydroxylates *N*-acetylmuramic acid residues leading to the production *N*-glycolylmuramic acid. The modification of peptidoglycan by NamH was determined to confer lysozyme resistance in *Mycobacterium smegmatis* [59]. It is likely that NamH confers lysozyme resistance to Mycobacterial species through the generation of *N*-glycolylmuramic acid, as NamH is well conserved in Mycobacterial genomes. It is hypothesized that *N*-glycolylmuramic acid residues may stabilize the cell wall; however, the mechanism of resistance is not fully understood [193]. However, recent work suggests that the presence of an *N*-glycolyl group blocks lysozyme from accessing the β-1,4 peptidoglycan bonds, preventing the muramidase activity of lysozyme and leaving the cell wall intact [59].

### 3.3. Alterations to Membrane Order

Apart from AMP repulsion and AMP target modifications as mechanisms of resistance, other changes in membrane composition can also reduce the susceptibility of bacteria to AMP-mediated killing. Alterations in Gram-positive membrane composition appear to contribute to AMP resistance by affecting the peptide interactions with the cell membrane. In particular, the degree of membrane fluidity appears to be an important determinant of AMP susceptibility. 

One example of a membrane alteration that confers AMP resistance is the saturation of membrane fatty acids. Investigations into the cell membrane components of nisin-resistant *L. monocytogenes* showed that some resistant strains contained a higher proportion of saturated (straight chain) fatty acids versus unsaturated (branched chain) fatty acids [194,195]. Additionally, a nisin resistant strain of *L. monocytogenes* produced lower concentrations of the lipid head group phosphatidylglycerol and less diphosphatidylglycerol than a nisin-susceptible strain [194,195,196]. This nisin-resistant strain also contained higher concentrations of the lipid head group, phosphatidylethanolamine, while the anionic membrane component, cardiolipin, was decreased [197]. These studies suggest that higher concentrations of saturated fatty acids, a decrease in phophatidylglycerol and an increase in phophatidylethanolamine head groups in the *Listeria* membrane lead to a decrease in cell membrane fluidity [194,195,196,197]. It is proposed that the decrease in membrane fluidity increases nisin resistance by hindering nisin insertion into the membrane [197].

The addition of other membrane components can also increase rigidity and lead to resistance to host AMPs and daptomycin in *S. aureus* [198]. Increased membrane rigidity in some Gram-positive organisms can result from carotenoid overproduction [199,200]. Carotenoids are organic pigments made of repeating isoprene units that are produced by plants, bacteria, and fungi [201]. Carotenoids, such as staphyloxanthin made by *S. aureus,* can stabilize the leaflets of the cell membrane by increasing order in the fatty acid tails of membrane lipids and lead to decreased susceptibility to AMPs [199,202,203]. This stabilization of fatty acid tails leads to an increase in cell membrane rigidity, which is suggested to limit insertion of AMPs into the membrane [204,205]. 

Though a higher concentration of saturated fatty acids in the membrane confers AMP resistance in some bacteria, other bacteria increase unsaturated fatty acid concentrations to increase resistance. In *S. aureus*, increased levels of unsaturated membrane lipids increase the resistance to the host AMP, tPMP (thrombin-induced platelet microbicidal proteins) [206]. Unsaturated fatty acids contain double bonds along the length of their carbon chain, which causes lipid disorder, thereby increasing membrane fluidity and impacting resistance to antimicrobials [206,207]. Other studies in AMP resistance found that methicillin-resistant *S.aureus* isolates that developed resistance to daptomycin also had increased resistance to host tPMPs and the human neutrophil peptide, hNP-1. These co-resistant strains have a phenotype defined by increased cell wall thickness and increased membrane fluidity [198]. It is hypothesized that these altered membrane arrangements may prevent efficient AMP insertion into the membrane [198,206,207]. 

At present, there is no clear explanation as to how alterations in membrane fluidity or rigidity lead to AMPs resistance. From the examples discussed above, it could be argued that the degree of fluidity required for resistance to a particular AMP may be as varied as the structures of the AMPs themselves, or perhaps is constrained to groups with similar mechanisms of action.

## 4. AMP Efflux Mechanisms

Transport, or efflux, is a common mechanism used by Gram-positive bacteria for the removal of toxic compounds and antimicrobials from cells. The majority of antimicrobial peptide efflux mechanisms consist of multi-protein ABC (ATP-binding cassette) transporter systems, which use ATP to drive the transport of substrates across or out of the cell membrane [208]. There are three primary types of ABC transporter systems implicated in Gram-positive AMP resistance: three-component ABC-transporters, two-component ABC-transporters, and single protein multi-drug resistance transporters, or MDR pumps [209]. All ABC-transporters are composed of two distinct domains: the transmembrane domain (permease) and the nucleotide-binding domain (NBD), which facilitates ATP-binding [209]. A less common efflux mechanism that has been identified is the Major Facilitator (MFS) Transporter module, which facilitates small solute transport via a chemiosmotic ion gradient [210]. This section will present the key types of AMP transporters found in Gram-positive bacteria and highlight the AMP resistance characteristics of these systems. 

### 4.1. Three-Component (LanFEG) Transporter Systems

Three-component ABC transporters, or LanFEG systems, are best characterized in AMP-producing bacteria. LanFEG systems are members of the ABC-type 2 sub-family of transporters, and consist of one protein with a nucleotide-binding domain (LanF) and two distinct transmembrane permeases (LanE and LanG) [211]. The majority of the characterized LanFEG systems are self-immunity mechanisms that provide protection against bacteriocins (typically lantibiotics) made by bacteriocin producer strains [38,112] (Table 1). The LanFEG transporters are often found in conjunction with LanI membrane-associated lipoproteins that can function in tandem with the transporter to provide greater resistance to AMPs [112,212,213]. 

The best-characterized LanFEG transporters are the NisFEG and SpaFEG systems found in strains of *L. lactis* and *B. subtilis* that produce the lantibiotic AMPs nisin and subtilin, respectively. Both NisFEG and SpaFEG provide resistance to their cognate substrates, but full resistance is achieved in concert with their associated substrate-binding lipoproteins, NisI and SpaI [100,213,214,215]. Immunity to the lantibiotic nukacin ISK from *Streptococcus warneri* does not involve a LanI protein, but instead contains a distinct membrane-associated protein termed NukH [96,216]. In contrast to the LanI proteins, NukH is not a lipoprotein; however, NukH does appear to function as a substrate-binding partner to the NukFEG transporter. Similar to LanI, NukH confers partial immunity to nukacin ISK, but full immunity requires the complete NukFEGH system [216,217]. 

Most characterized LanFEG systems confer resistance only to the AMP made by a producer strain, although examples have been identified that provide resistance to multiple AMP substrates in non-producer bacteria. In *Clostridium difficile*, the CprABC transporter (a LanFEG ortholog) confers resistance to nisin, gallidermin, and likely other structurally dissimilar lantibiotic peptides [85,86]. The regulation of immunity and AMP biosynthetic genes are typically coupled in bacteriocin producer strains [112]. The ability of the CprABC system to confer resistance to multiple unrelated peptides may result from the uncoupling of the immunity mechanism from bacteriocin synthesis. But non-producers that have immunity genes in the absence of AMP biosynthetic operons can have relaxed substrate specificity that allows for recognition of multiple bacteriocins. Thus, Lan transporter cross-immunity to multiple AMPs could provide a significant competitive advantage to non-producer bacteria. Indeed, a homology search for LanFEG proteins reveals that the genomes of many other Firmicutes encode predicted bacteriocin transporters that are not coupled with apparent bacteriocin synthesis genes. Hence, like other antibacterial resistance mechanisms, the LanFEG systems have found their way into non-producing species [85,86]. 

### 4.2. Two-Component ABC-Transporter Systems

Two-component ABC-transporters make up the majority of transporter-mediated AMP resistance characterized in non-AMP producing bacteria. The canonical two-component ABC-transporter consists of one nucleotide-binding protein and a separate membrane-spanning permease [218,219]. Unlike most LanFEG systems, two-component transporters often provide resistance to multiple AMPs and are common among Gram-positive bacteria. As outlined in Table 1, numerous examples of these transporters have been identified that can provide resistance to AMPs produced by humans and bacteria, including cyclic peptides and some non-peptide antibiotics [218,220]. 

There are two main types of two-component ABC-transporter systems that confer resistance to AMPs among Gram-positive bacteria. The first and most common type is often referred to as the BceAB group [218,221]. BceAB transporter systems contain an archetypal ATP-binding protein of about 225–300 amino acids and a larger permease component that ranges in size from 620–670 amino acids. The prototype of this transporter group, BceAB, was first identified as a bacitracin resistance mechanism in *B. subtilis* [67,68]*.* Since the identification of BceAB, dozens of similar transporters have been discovered in pathogenic and non-pathogenic Gram-positive species, including *S. aureus*, *L. monocytogenes*, *S. pneumoniae*, and *L. lactis* (see Table 1 for examples) [62,71,77,80]. Members of the BceAB group have demonstrated resistance to a wide-range of bacteriocins, mammalian and fungal defensins, peptide antibiotics, and other antimicrobial compounds (Table 1). Although many of the BceAB transporters confer resistance to AMPs *in vitro*, the roles of these transporters in the virulence of pathogenic species are not known.

Another common type of a Gram-positive ABC-transporter that confers AMP resistance is the BcrAB(C) system. The BcrAB(C) transporter confers resistance to bacitracin and was originally identified in a bacitracin producer strain of *Bacillus licheniformis* [81]. BcrAB transporters can be distinguished from the BceAB systems by size and topology: BcrA is an ATP-binding cassette that ranges from about 280–320 amino acids, while the BcrB permease modules are smaller, at approximately 200–250 amino acids. BcrAB is often encoded with a third protein, BcrC (or BcrD), which allows for higher resistance to bacitracin than the BcrAB transporter alone [81,222,223]. Initially it was hypothesized that BcrC functioned as part of the BcrAB ABC-transporter, however it was later demonstrated that BcrC acts as an undecaprenyl pyrophosphate (UPP) phosphatase that competes with bacitracin for UPP [222]. The BcrAB transporters are predicted to be structurally similar to the LanFEG transporters, though the Lan systems function through two dissimilar permease components, while Bcr systems operate with only one permease subunit (BcrB) [38,82,218]. Aside from the bacitracin producer strains, BcrAB and orthologous transporters have been shown to confer resistance to bacitracin in many strains of *E. faecalis*, as well as some *Streptococcus* and *Clostridium* species [35,82,83,224].

### 4.3. Single Membrane Protein Antimicrobial Transporters

Multi-drug resistance (MDR) ABC-transporters are common bacterial mechanisms of resistance to peptide and non-peptide antibiotics [225]. Though these transporters are most common among characterized mechanisms for non-peptide antimicrobial resistance in Gram-positive bacteria, there are examples of MDR transporters that confer resistance to AMPs. One notable MDR AMP resistance mechanism consists of the LmrA/B proteins encoded by some *L. lactis* strains [60,226]. A LmrA MDR efflux pump was first described in a non-producer strain of *L. lactis* [226]. LmrB is an ortholog of LmrA found in *L. lactis* strains that produce the bacteriocins LsbA and LsbB [60]. LmrA/LmrB are membrane proteins with six predicted transmembrane segments and a C-terminal, nucleotide-binding domain [60]. LmrA provides broad resistance against a long list of peptide antibiotics and cytotoxic compounds, while LmrB confers resistance to the two bacteriocins LsbA and LsbB [60,226]. A BLASTp homology search revealed the presence of additional orthologs of LmrA/B encoded within the genomes of hundreds of Gram-positive Firmicutes, though the function and significance of these remains unknown.

A less common type of single-protein transporter involved in antimicrobial peptide resistance is exemplified by the QacA transporter of *S. aureus* [61]. QacA is a member of the major facilitator superfamily (MFS) of membrane transport proteins, which use proton motive force, rather than ATP, to drive the efflux of substrates [227]. QacA confers resistance to a variety of toxic dyes, antiseptics and disinfectants [228,229]. In addition to cationic toxins, QacA provides resistance to thrombin-induced platelet microbicidal protein (tPMP), a host-derived antimicrobial peptide [61]. QacA-dependent tPMP resistance was found to confer a survival advantage in an animal model of infection, and increased resistance to tPMP in *S. aureus* also correlates with endocarditis in humans [61,230]. QacA orthologs have also been identified in other staphylococci, as well as in *Enterococcus* and *Bacillus* species, though the ability of these orthologs to transport AMPs is not understood [231,232]. 

## 5. Conclusions 

Antimicrobial peptides are diverse in both structure and function and are produced by all forms of life. As such, AMPs are an ancient defense mechanism, and resistance mechanisms to AMPs have been selected for as long as AMPs have existed. Gram-positive bacteria are ancient producers of AMPs and as a consequence, these organisms likely developed some of the first AMP resistance mechanisms. 

Herein we have detailed a wide variety of AMP resistance mechanisms found in Gram-positive bacteria (summarized in Figure 1). AMPs resistance mechanisms can be broad spectrum, such as MprF and the Dlt pathway which function by decreasing the net negative charge of the bacterial cell surface, thereby reducing the attraction for positively charged AMPs from the cell. Conversely, AMP resistance mechanisms can be highly specific and only confer resistance to a single peptide. AMP resistance mechanisms can be confined to a particular species or genus, such as NamH in *Mycobacterium*, or can be distributed among multiple species, such as the LanFEG systems. AMPs resistance mechanisms are dynamic; they can be passed from species to species via bacteriophages or horizontal gene transfer, and can change specificity and function over time through evolution [85,86,105,233]. Under selective pressure, AMP resistance mechanisms can evolve to suit the needs of a particular species in its own niche [234]. 

At present, many AMPs are being investigated as potential antimicrobial therapies [235,236,237,238,239,240]. AMP drug development should be carefully vetted because like any naturally-produced antimicrobial, cognate resistance mechanisms for AMPs are already present in the producer bacterium. While these resistance mechanisms may be found more frequently in producer strains, each has the propensity to be passed on to other genera or species within a shared environmental niche. Because the presence of AMPs provides high selective pressure for the acquisition of resistance, it is important to consider the potential for resistance mechanism transfer between bacteria when developing AMPs for clinical use [241,242]. Additionally, depending on the AMP resistance mechanism that is selected for, a multitude of issues may arise if the mechanism of resistance is broad-spectrum. A broad-spectrum AMP resistance mechanism could restrict the already limited clinical treatment options for use against some Gram-positive pathogens and may undermine our own immune response by conferring resistance to our own innate immune system peptides [243].

Antimicrobial peptide resistance is not as well characterized for Gram-positive bacteria as it is for Gram-negative bacteria. Thus, it is likely that many more mechanisms of antimicrobial resistance remain to be discovered in Gram-positive species. As more AMPs are found, new Gram-positive AMP resistance mechanisms will undoubtedly be revealed.
